# Arctic sea ice melt leads to atmospheric new particle formation

**DOI:** 10.1038/s41598-017-03328-1

**Published:** 2017-06-12

**Authors:** M. Dall´Osto, D. C. S. Beddows, P. Tunved, R. Krejci, J. Ström, H.-C. Hansson, Y. J. Yoon, Ki-Tae Park, S. Becagli, R. Udisti, T. Onasch, C. D. O´Dowd, R. Simó, Roy M. Harrison

**Affiliations:** 1 Institute of Marine Sciences (ICM) Consejo Superior de Investigaciones Científicas (CSIC), Pg. Marítim de la Barceloneta 37–49, 08003 Barcelona, Spain; 2 Centre for Atmospheric Science Division of Environmental Health & Risk Management School of Geography, Earth & Environmental Sciences, University of Birmingham, Edgbaston Birmingham, B15 2TT United Kingdom; 3 School of Physics & Centre for Climate & Air Pollution Studies, National University of Ireland, Galway, Ireland; 4 Aerodyne Research, Inc., Billerica Massachusetts, USA; 5 Department of Environmental Science and Analytical Chemistry, Stockholm University, 10691 Stockholm, Sweden; 6 Korea Polar Research Institute, 26, SongdoMirae-ro, Yeonsu-Gu, Incheon Korea; 7 Department of Chemistry “Ugo Schiff”, University of Florence, Via della Lastruccia 3, 50019 Sesto Fiorentino, Florence Italy; 8 Department of Environmental Sciences/Center of Excellence in Environmental Studies, King Abdulaziz University, PO Box 80203, Jeddah, 21589 Saudi Arabia

## Abstract

Atmospheric new particle formation (NPF) and growth significantly influences climate by supplying new seeds for cloud condensation and brightness. Currently, there is a lack of understanding of whether and how marine biota emissions affect aerosol-cloud-climate interactions in the Arctic. Here, the aerosol population was categorised via cluster analysis of aerosol size distributions taken at Mt Zeppelin (Svalbard) during a 11 year record. The daily temporal occurrence of NPF events likely caused by nucleation in the polar marine boundary layer was quantified annually as 18%, with a peak of 51% during summer months. Air mass trajectory analysis and atmospheric nitrogen and sulphur tracers link these frequent nucleation events to biogenic precursors released by open water and melting sea ice regions. The occurrence of such events across a full decade was anti-correlated with sea ice extent. New particles originating from open water and open pack ice increased the cloud condensation nuclei concentration background by at least ca. 20%, supporting a marine biosphere-climate link through sea ice melt and low altitude clouds that may have contributed to accelerate Arctic warming. Our results prompt a better representation of biogenic aerosol sources in Arctic climate models.

## Introduction

The climate of the Arctic is changing faster than almost everywhere else on Earth, a phenomenon known as the Arctic amplification^1^. Current climate models show large differences in projected outcomes of present and future Arctic warming^2, 3^. Air quality regulations on emissions in the Northern Hemisphere, ocean and atmospheric circulation, and Arctic climate are inherently linked^4–6^. The Arctic has a pronounced aerosol annual cycle, with the haze period associated with high accumulation mode aerosol concentrations over the dark months^7, 8^. In the beginning of the summer daylight period, changes in the general circulation that limit continental and anthropogenic inputs, along with the increased presence of low-level clouds (and thus more effective wet removal of pollutants) result in fast atmospheric clean-up of the haze^9–11^. Lower aerosol loadings and increased photochemistry lead to a peak in ultrafine aerosols (<100 nm) during the Arctic summer^8^. The Arctic environment undergoes various changes with the potential to affect local and regional aerosol properties. Wet removal by snow or rain is the main sink for accumulation-mode particles, whereas condensation, cloud processing, and transport may be the main sources of these particles^12^. Multifaceted - yet poorly understood - atmospheric processes by which aerosols contribute to the numbers of cloud droplet condensation nuclei strongly affect the radiative balance^13^.

The formation of new particles in the atmosphere occurs regionally, but makes an important contribution to the worldwide aerosol particle number concentrations^14^. Within the Arctic, such events are suggested to be formed via secondary aerosol formation and being of marine biological origin from the open waters between ice floes^15–19^. Other studies point to an alternative hypothesis involving fragmentation and/or dispersion of primary marine polymer gels also originated in water next to the ice^17, 20, 21^.

Overall, our understanding of climate-relevant aerosol sources, formation processes and size distributions in the remote Arctic region remains incomplete, in part due to the scarcity of long term observations and appropriate methodologies for data interpretation^22–25^. Here we applied clustering analysis to a decade-long record of aerosol numbers and size distributions obtained at the Arctic monitoring site of Mount Zeppelin in Svalbard. The main objectives were (a) a categorization and quantification of the aerosol ultrafine population; (b) the association of such aerosol categories with air mass back trajectories and geographical and environmental span; (c) the apportionment of natural and anthropogenic aerosol sources by correlation with chemical markers, and (d) the impact of the elucidated aerosol categories to the Cloud Condensation Nuclei (CCN) population, with particular emphasis at the new particle formation events.

## Results

### Categorising Arctic ultrafine aerosols and new particle formation events

**Figure 1:**
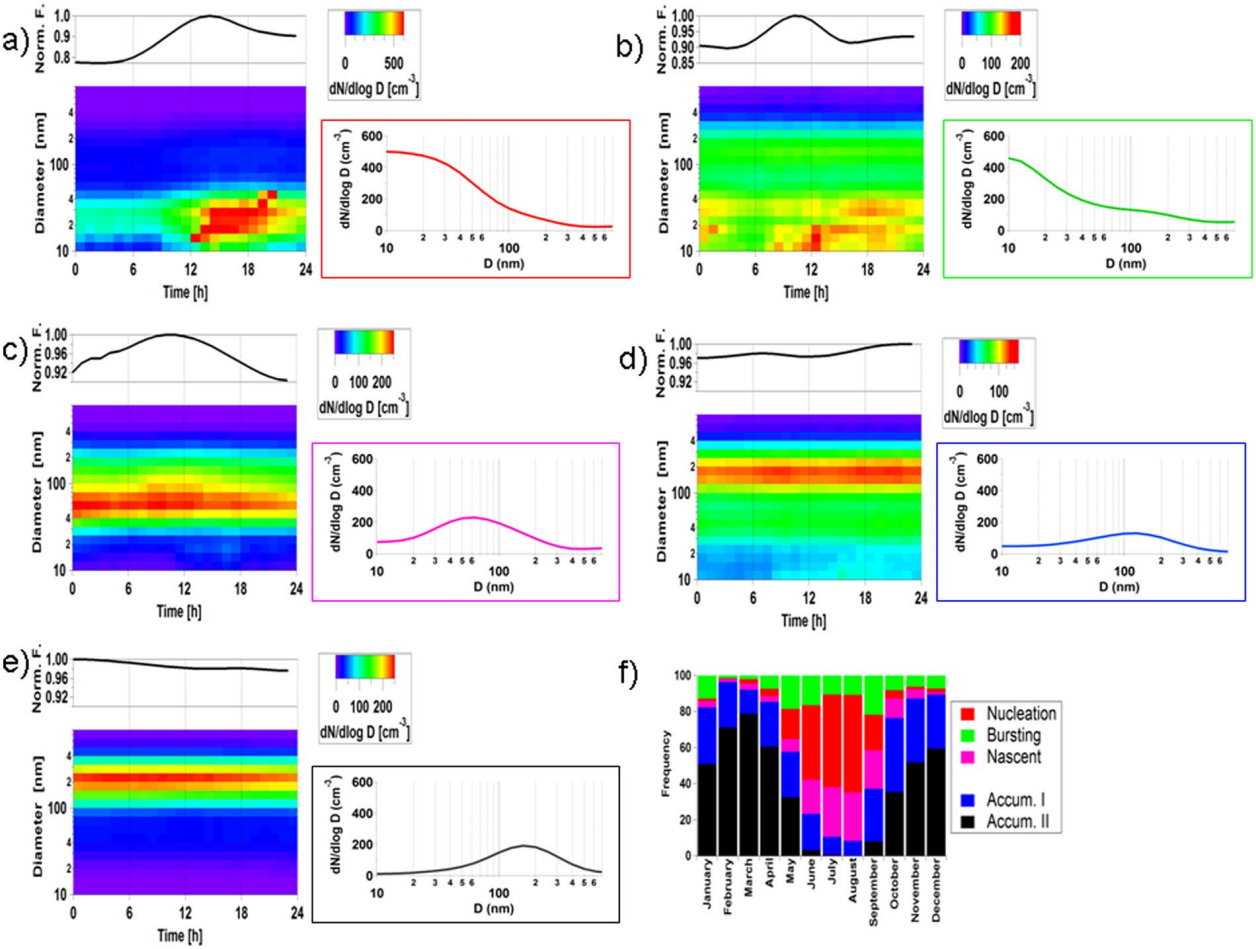
(**a–e**) Daily aerosol size distributions cluster results (bottom), average Daily N_10–500_ particle number concentration (top) and average daily size distribution (on the right coloured panel); and (**f**) annual frequency distributions of the five aerosol categories. New particle formation events were also validated by N_3–10nm_ concentrations (measured by tandem particle counters with lower detection limits of 3 and 10 nm, respectively; see methods) of 316 ± 110, 168 ± 58, 128 ± 44, 73 ± 25, 99 ± 36 cm^−3^ for clusters (**a–e**), respectively.

We executed K-means cluster analysis (see Methods) of particle number size distributions using 73,000 hourly distributions collected over 11 years (2000–2010, 84% data coverage^8^). Here, we refer to ultrafine as particles with diameters between 10 and 100 nm. Data were clustered at daily averaged resolution; in total, we classified five categories (Supporting Information), three of which clearly described the ultrafine aerosol population (Fig. 1).

- *“Nucleation”* ultrafine. Occurring annually 18% of the time, Fig. 1a shows a daily aerosol evolution starting in the morning at smallest-detectable sizes (10 nm) and reaching 60–80 nm in the late afternoon. Its diurnal profile peaks at 14:00–15:00. It presents a peak in summer (July–August, 51%, Fig. 1f); overall 95% of these events were detected during daylights months. The name of this category - which will be used below - stands for continuous gas-to-particle growth occurring after the particle nucleation^14^ (Figure S2a).
- “*Bursting*” ultrafine. Less frequent (11%), it shows an aerosol mode in the smallest detectable sizes at 10 nm (Fig. 1b). Its daily trend shows a peak in the morning at 10:00–12:00, although on average other minor aerosol bursts can also be seen during evening and night time. The annual trend (Fig. 1f) shows an enhancement during the season transition periods in late spring (May) and early autumn (September); overall, 65% of these events were detected in daylight months. The name of this category refers to an aerosol population that bursts and begins to exist or develop, but fails to grow to larger sizes like in the *nucleation* category (see Figure S2b).
- “*Nascent*” ultrafine. It occurs annually 12% of the time, with a broad Aitken mode centred at 55 nm without a clear diurnal pattern (Fig. 1c–f). The name of this category emerges from growing ultrafine aerosol resulting from the processing of local and regional marine aerosols, a phenomenon previously described to occur mainly in summer^8, 26^.

The two remaining clusters (“*Accumulation I” and “Accumulation II”*) were characterized by larger size modes that contributed little to the overall ultrafine aerosol population numbers (Fig. 1d,e). In summary, mainly “*Bursting*” and “*Nucleation*” aerosol categories contribute to NPF events.

### Elucidating source regions

A number of regions contribute aerosols to the Arctic boundary layer, depending on the measurement site^25^. The mountainous site Zeppelin^8, 27^ receives long-range transported pollution predominantly from Eurasia during winter and spring. In summer, Zeppelin is often located south of the Polar Front and thus receives mainly marine air masses from the Atlantic Ocean. Apportioning the origin and trajectory of the air masses is critical - therefore - to interpret locally monitored aerosol ultrafine events. In order to associate our aerosol categories to air masses and surface characteristics, a cluster analysis of air mass back trajectories arriving at Zeppelin every 6 hours during the 11 year record (see Supporting Information, Figure S3) was carried out; resulting in five main air-mass clusters (Table S1). The most frequent air mass (cluster 5, 27%) was stagnant air from the South, whose occurrence peaked in summer (38–45%). The second most frequent air mass (cluster 4, 25%) came from the East, mainly during winter (33–36%). Minor clusters included South West and Greenland (cluster 3, 19%), North (cluster 2, 15%) and Northern long distance (cluster 1, 14%). We subsequently matched the air mass classification analysis with our aerosol k-means cluster analysis. Table S1 - obtained from merging analysis presented in Figs 1 and Fig. S3 - shows the emerging seasonal associations, with ultrafine aerosol categories mainly associated with air masses from the South/South East sector (63–65%).

**Figure 2:**
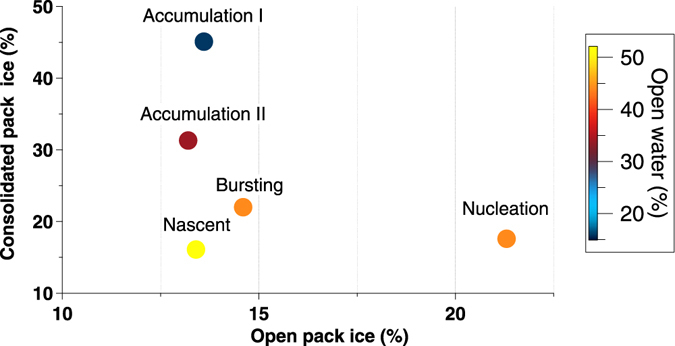
Percentages of total time (hours) of air mass back trajectories travelling over different sea ice areas for each of the 5 aerosol categories.

In a further analysis, we calculated how far each air mass travelled over zones distinguished by their surface characteristics, namely land only, land covered by snow, sea ice and open water (Methods, Table S1). Ultrafine aerosols did not seem to be frequent when air masses travelled over land (3–7%), or land covered by snow (13–17%). By contrast, the majority of the ultrafine aerosols were associated with air masses travelling over open water (43–51%) and sea ice (29–39%). Arctic sea ice is a spatially complex physical environment - a vast biome composed of multiple habitats such as the upper and lower ice surfaces, snow cover, brine channels, melt ponds, ice openings and ice floes of all sizes, and the surrounding sea water. Changes in the amount of ocean surface covered by ice play an important role in the global climate system^28, 29^. Forthcoming definition of sea ice regions^28^, we classified “consolidated pack ice” as regions with pack ice concentration higher than 80%, “open pack ice” as regions with sea ice concentration higher than 15% and lower than 80% within the consolidated ice region, and “open water” as regions with sea ice concentrations lower than 15%. For each day of each aerosol category, we calculated the amount of time spent by the associated air mass trajectory over the sea ice regions. Results are summarised in Fig. 2. *Nascent* ultrafine is dominated by air masses travelling over open water (52%), whereas *accumulation* I and II over consolidate packed sea ice (31–45%). *Bursting* ultrafine seems to have intermediate undefined conditions. Figure 2 shows that the *nucleation* category is associated with air masses travelling over open pack ice. Even though both categories *nucleation* and *nascent* peak in summer (Fig. 1f), the former is the only one mostly associated with open pack ice relative to consolidated pack ice. Thus, areas with melt ponds, open leads amidst the pack ice, and variable ice floe percentages across the open pack ice, all seem to be strong sources of precursor gas phase species responsible for aerosol new particle formation and growth in the summer Arctic. Our 11 year record supports previous short studies conducted during icebreaker expeditions at high Arctic locations^17^.

The ultrafine aerosol categories detected in this study were recorded in the planetary boundary layer. The vertical distribution of airborne particles over the summer Arctic is governed by the complicated interplay between local sources, air mass origin and long-distance transport, mixing events and cloud processing. Previous observations showed new particle formation events taking place at high altitudes including the free troposphere^30^ as well as in the boundary layer near the surface^31^. Recent vertical profiles have revealed the latter as a more plausible and common source^32, 33^. The absence of deep convection limits the entrainment of free tropospheric aerosols into the inversion layer. Therefore, local natural sources, in combination with boundary layer transport of precursor gases from the open pack sea ice region, are thought to be significant contributors to the detected aerosol population. Further information on chemical and physical aerosol properties are presented in the next section.

### Anthropogenic and biogenic chemical markers

**Figure 3:**
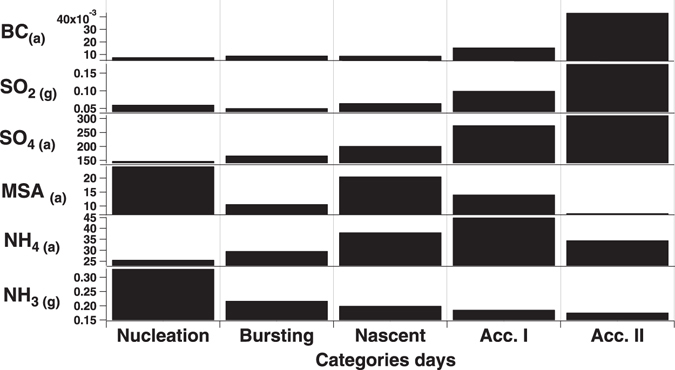
Average daily concentrations of selected chemical tracers for each aerosol category.

It is still debated as to how different natural and anthropogenic sources contribute to the Arctic aerosols^8, 34^. The Arctic is subject to a strong anthropogenic influence during winter. Indeed, the highest daily concentrations of anthropogenic equivalent black carbon (EBC) were detected during days classified into the two *accumulation* categories (43 ng m^−3^ and 16 ng m^−3^, respectively), which are more frequent in winter (Fig. 1f and 3). By contrast, the remaining three aerosol categories describing the ultrafine population were recorded during periods with the lowest black carbon concentrations (7–9 ng m^−3^, Fig. 3). SO_2_ in the Arctic has both anthropogenic and natural sources^9^, but in our study it paralleled the EBC pattern (r = 0.98, N = 2850 days, Fig. 3). Sulphate, conversely, showed a lower correlation to EBC (r = 0.71, N = 140) and no clear association with any of the aerosol clusters. Non sea salt Sulphate in the Arctic atmosphere originates from anthropogenic and biogenic sources, including dimethylsulphide (DMS) and methane sulfonate (MSA)^35, 36^. Notably, we found the highest daily concentration of MSA associated with nucleation event days over summer (Fig. 3). A correlation between summertime particle number concentrations and MSA concentrations was previously found^15, 16, 18^. However, associations between DMS flux, changes in sea ice extent and phytoplankton productivity are not fully understood. Sea ice is an organic sulphur-rich environment because ice algae generally contain high intracellular levels of DMSP for osmoregulation and cryoprotection^22^. Even though surface-ocean DMS and chlorophyll *a* concentrations are typically not correlated over large spatial and temporal scales, recent studies suggest that the variability in the DMS mixing ratios in Svalbard air may be explained by the variability in the chlorophyll *a* concentration in the vicinity of the islands^35, 37, 38^.

Another ion commonly found in Arctic aerosol is ammonium. Average daily concentrations for the five aerosol categories show that this compound paralleled sulphate (r = 0.85, N = 140) but not gaseous ammonia (r = −0.66, N = 135) across the aerosol clusters (Fig. 3). Interestingly, ammonia gas concentration was the highest (0.33 μg N m^−3^) during nucleation event days relative to all other days (0.18 ± 0.11 μg N m^−3^; Fig. 3). In remote marine environments, the ocean is thought to be the dominant source of NH_3_ by zooplankton excretion and bacterial remineralization of phytoplankton-derived organic matter^39^. New comparisons of model and field data lead to a lower estimate of the global marine emission than previously suggested^40^, although there remains considerable uncertainty^41^. In the Arctic, it has been suggested that that ice melting is a significant source of ammonium^42^. Protein-like compounds accumulate at the sea-ice interface when the sea ice melts^43^, and protein-like organics of marine origin are a substantial component of High Arctic Aerosols^44^. The most labile of the ice-released protein-like material will be quickly degraded into volatile methylamines by marine bacteria^39^, thus favouring gaseous emission fluxes from ice-pack openings and the ice edge. This is likely similar to what occurs with sulphur^23^. Ammonia has also been found enriched in Antarctic ice relative to surface seawaters, suggesting that ice melt is a significant source, likely as a consequence of biological activity within the ice^45^. There is strong evidence that coastal seabird colonies are sources of NH_3_ in the summertime Arctic^46^, although there is still significant uncertainty^47^. Recently, ammonia from seabirds was found to be a key factor contributing to bursts of newly formed coastal particles at Alert, Canada^48^. A pan-Arctic aerosol number size distribution study revealed that there is no single site that can be considered as fully representative for the entire Arctic region with respect to aerosol number concentrations and distributions^49^. Our air mass analysis excluded local influence of coastal sources on detected NPF events over 11 years. Further studies are needed to elucidate and quantify different particle sources including natural seabird colonies and open pack sea ice emission.

The chemical composition of the categorized Arctic ultrafine aerosol is not known at this stage. Recent work in the study region^50^ reported iodine oxoacids and iodine oxide vapours driving the formation and initial aerosols growth process. Involvement of ammonia and amines in particle nucleation at mid-latitudes has become well established^19^, and recent measurements suggest that local ammonia sources in the summer Arctic are sufficient to promote particle formation^46–48^. Whilst we cannot directly link any compound to observed newly formed particles, our aerosol chemistry correlation data suggest a crucial role of biogenic precursors originating over sea-ice regions in the Arctic in ultrafine aerosol formation.

Primary emissions of biological particles (microgels) from the ocean^20–22^ may explain part of the aerosol concentrations of the category *bursting*. However, part of the diurnal profile may also be explained by secondary new particle formation bursts. The absence of particle growth for this category may depend upon a delicate balance between a high vapour formation rate and a low condensation sink^14^. Therefore, calculation of the Condensation Sink (CS, see methods) for the five different aerosol categories may help our interpretation. Indeed, the average CS for the different categories (Figure S4) shows that *bursting* have among the highest CS values, suggesting such a competing process is probably limiting aerosol growth. Conversely, the diurnal profile of the summer-dominant *nucleation* category strongly indicates that these aerosols resulted from new particle formation (NPF) events through photochemical reactions of gaseous precursors of biogenic origin. Thus, secondary aerosols seem to be the prevailing - but not sole^22, 26^ - mechanism in governing ultrafine aerosol numbers in the Arctic. *Nascent* ultrafine likely result from a combination of marine processes^26^. Figure S4 brings additional support to our finding, stressing the importance of biogenic precursors released by open water and melting sea ice (Fig. 2). Category *nascent* possesses the lowest CS values and - regardless of occurring mainly during summer time (Fig. 1f) - the absence of gas precursors is likely the reason for the absence of small ultrafine aerosol in this category.

### Nucleation events, sea ice and implications for climate

Despite the presence of the ice cover and extreme light and temperature conditions, the Arctic shelf seas rank amongst the most biologically productive in the world. As the region warms^1^, decreasing sea ice alters marine ecosystems by, e.g., increasing the rates of phytoplankton net primary production by 20–30%^51^. Therefore, the enhanced source strengths of primary organic biological particles and the aerosol precursors DMS and nitrogen volatiles are likely to increase the oceanic influence on atmospheric composition^21, 52^. Some studies have suggested an increase in biological activity with increased temperature and decreased sea-ice cover during summer, leading to elevated DMS production and emission^15, 18, 27, 38^. However, modelling results^25^ suggest that increased summertime DMS emissions will not cause a strong climate feedback due to the efficient removal processes for aerosols. Such results are highly dependent on aerosol arising from the microbial cycling of sulphur, which still is largely unresolved^23^.

**Figure 4:**
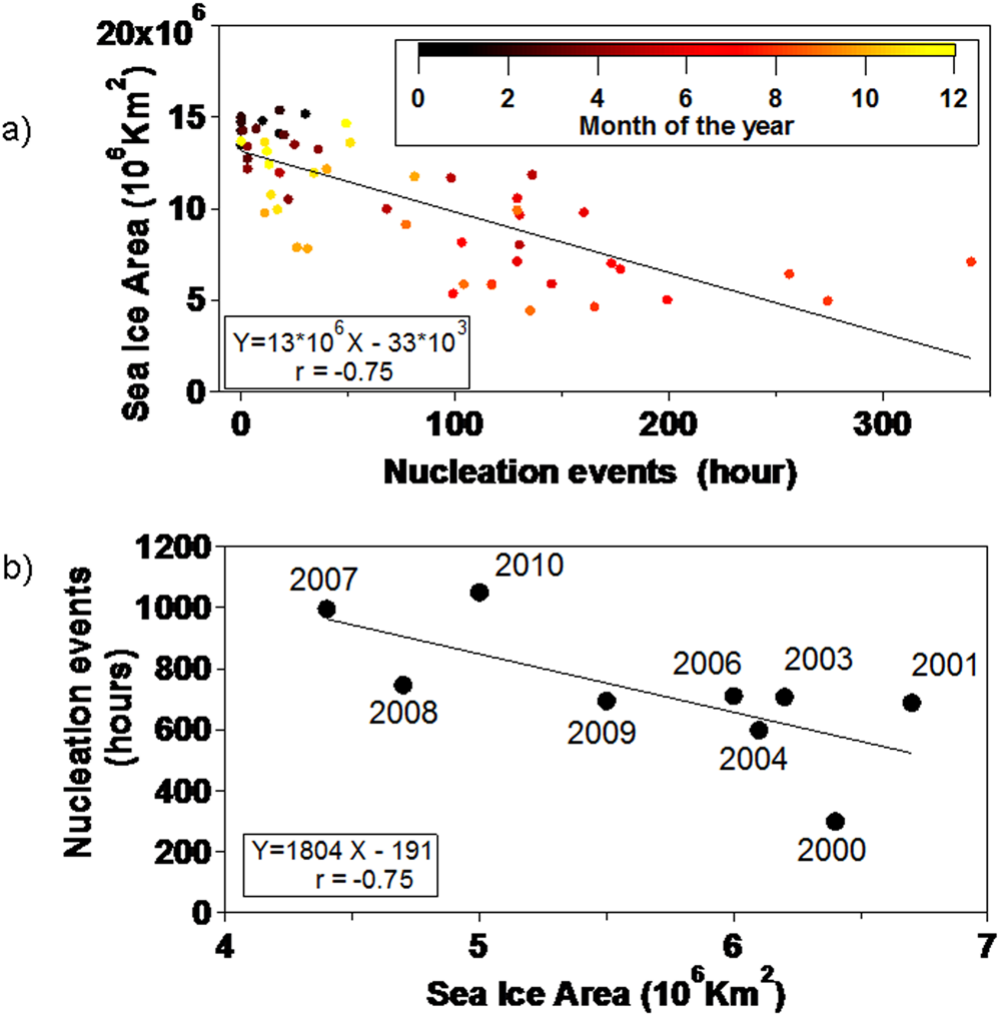
Relationship of sea ice extent (calculated over nearby regions of Greenland Sea and Barent Sea) with the *nucleation* aerosol category. Year 2002 and 2005 are not considered given low DMPS data capture^8^.

We wanted to test whether nucleation events observed throughout the 11-year period presented any trend with the recorded sea ice extent. Figure 4a shows a striking correlation of r = −0.75 between monthly sea ice extent and nucleation events. The correlation is found robust also at annual resolution (r = −0.75, Fig. 4b). Sea ice is the central component and most sensitive indicator of the Arctic climate system and, according to all available climate projections it will continue to decrease. This study suggests that as sea ice pack extent retreats, either every summer or in the longer term with global warming, biogenic productivity flourishing in the open-ocean and marginal sea ice zones is responsible for increased new particle production.

**Figure 5:**
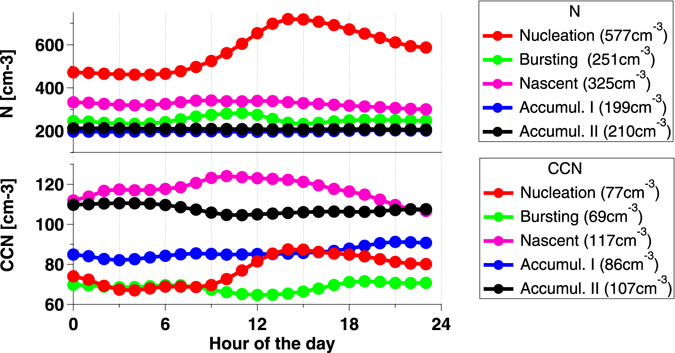
Diurnal profiles of N and CCN categorised according to aerosol size resolved particle number distribution clustering. In brackets, average particle number and CCN concentrations for each aerosol cluster.

Clouds, and related climate feedbacks, are major sources of uncertainty in climate model projections, and the Arctic is not an exception. Even small changes in cloud cover and albedo may have a strong impact on the energy balance at the surface and hence on the summer ice-melt^53^. The radiative properties of summertime liquid clouds are very sensitive to the number of cloud condensation nuclei (CCN), which in turn is very sensitive to the local formation of new particles when aerosol long-range transport is limited^23, 54^. NPF strongly controls the number of particles ≤ 200 nm in all seasons, and particularly in summer^12^. NPF involves the construction of molecular clusters and the subsequent growth of these clusters to larger sizes. In order to see if the recorded nucleation events had an effect on the overall aerosol numbers and CCN concentrations, we hourly-resolved total particle number counts (N) and CCN data collected over the period 2007–2010 (1,219 days). Figure 5 shows that daily NPF events are responsible for the highest ultrafine particle numbers, overall contributing 37% of the detectable particles, up to 75% in the summer months. To affect cloud radiative properties, these small particles had to grow to CCN sizes. Indeed, the CCN baseline number increases by 21% (from 70 ± 5 cm^−3^ to 86 ± 5 cm^−3^) during NPF events (Fig. 5). It is not known at this stage if the high levels of particle number concentrations detected in *Nucleation* days (highest background N at about 500 cm^−3^ at about 09am) will grow to CCN sizes.

## Discussion

The Arctic is a complex environment where, as opposed to large parts of the world, cloud properties together with bright ice and snow-covered surfaces cause clouds to actually warm the surface by more efficiently trapping a portion of the outgoing long-wave radiation. Various studies indicate that Arctic cloud cover has increased during recent decades^55^. Greater cloudiness in this region could lead to warming and accelerated sea-ice melt. Subsequent changes in the moisture source likely result in changes in precipitation that further complicate the energy balance, creating notable uncertainties for climate predictions^56^. New sources of particles and CCN are expected from increased anthropogenic activities in the ice-freer Arctic in coming years^55^. In order to evaluate their potential impact on Arctic climate, it is necessary to better understand and quantify the natural phenomena behind ocean-ice-aerosol-cloud interactions, upon which anthropogenic aerosols exert radiative forcing^24^.

**Figure 6:**
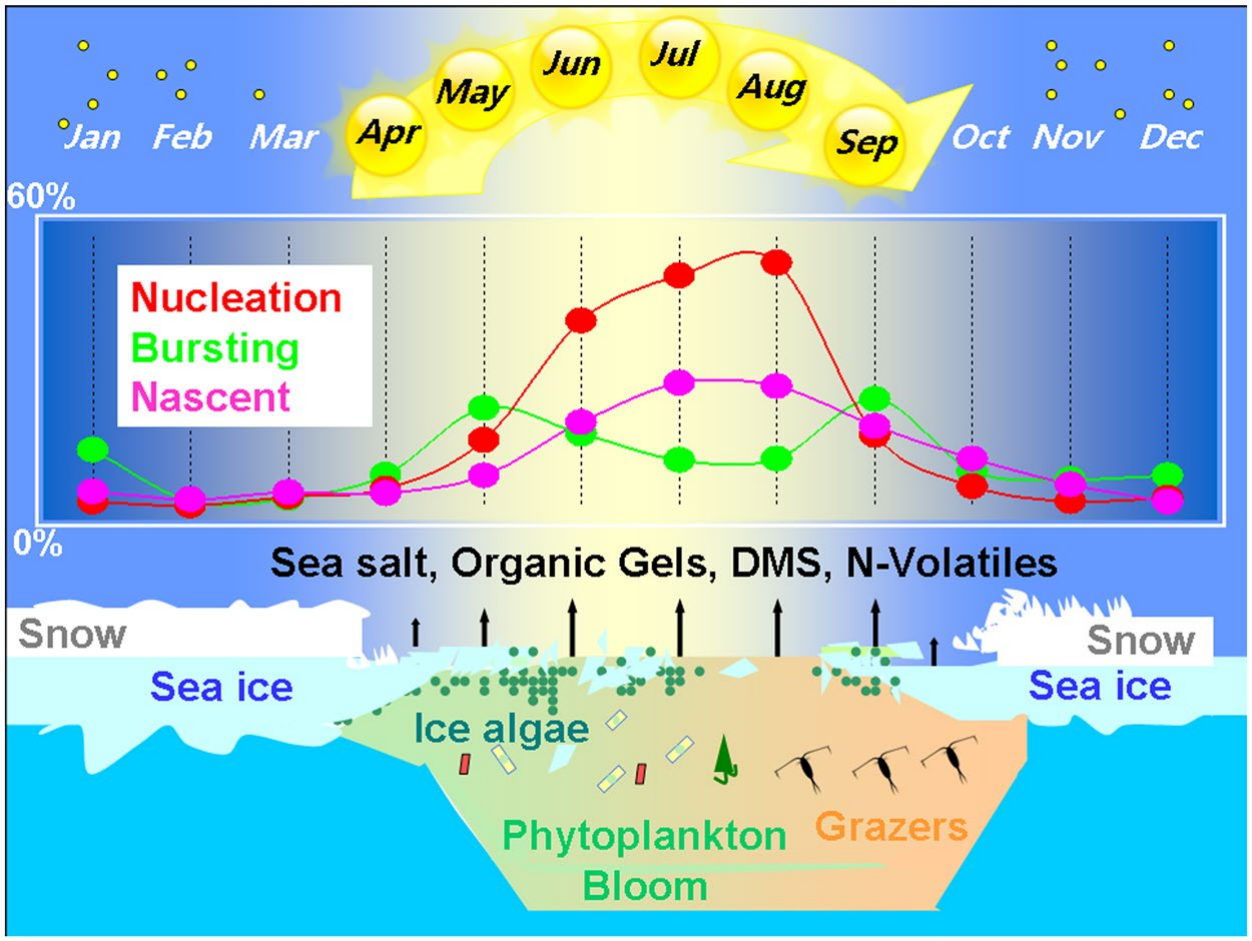
Schematic illustrations of the seasonal cycle of sea-ice, microbiota, sea-to-air emissions and ultrafine aerosols in the Arctic. Aerosol size ranges for the aerosol categories are 10 ± 2 nm, 32 ± 12 nm and 50 ± 11 nm for Nucleation, Bursting and Nascent ultrafine, respectively. Both Bursting and Nucleation aerosol categories contribute to new particle formation events.

We conclude with a schematic illustration of the seasonal sea-ice and aerosol cycle (Fig. 6). Our observations indicate that marine biogenic emissions are responsible for about 20% increase in particles that will nucleate droplets at supersaturations of 0.4% or lower over the Arctic. As sea ice retreats and thins, it opens the ocean to increased solar radiation and wind exposure. Increased primary production is enhanced through sea ice melt ponds and ultimately though the water column upon sea ice breakup. During summer, favourable conditions (e.g. sources of biogenic gaseous precursors, photochemical activity and low condensation sink) create new particles that reach CCN sizes via secondary gas to particle *nucleation and growth* mechanisms. In spring and autumn, conversely, ultrafine particle bursts are hardly accompanied by further growth, probably because of a lack of gas phase precursors. Despite this general scheme of temporal evolution, the Arctic ocean-sea ice-atmosphere coupled system remains largely unexplored but reveals highly nonlinear and spatially heterogeneous processes. Further integrated studies with joint multi-component observations are warranted.

## Methods

### Location

The aerosol number size distribution observations that are presented in this study were collected at the Zeppelin observatory located on the top of Mt. Zeppelin, Svalbard (78 56 N, 11 30 E, 474 m above sea level), just outside the small community of Ny-Alesund. The station represents remote Arctic conditions.

### Aerosol size distribution cluster

Detailed information on closed-loop Differential Mobility Particle Sizer (DMPS) measurements can be found elsewhere^8^. In order to group together the number size distributions (NSDs) into common sets which were dependent mainly on the shape of the distribution and not the magnitude, the NSDs were normalised to the vector sum and cluster analysed using k-means clustering^57^. K-means clustering aims to partition the observations into k clusters in which each observation belongs to the cluster with the nearest mean. The analysis works given a predefined number of clusters k and an optimum needs to be decided upon. The optimum cluster number was derived using the Dunn Index and Sihouette Width (silwidth) Figure S1. The Dunn index (DI) is a function of the ratio of the minimum cluster separation to the maximum cluster, implying that the larger the Dunn index the more compact and well separated. High values of DI identify sets of clusters that are compact, with a small variance between members of the cluster, and well separated, where the means of different clusters are sufficiently far apart, when compared to the within-cluster variance^58, 59^. However, as the number of clusters increases there is a tendency of DI to decrease. A second useful measurement is the Silhouette width^60^, which is a measure of the similarity of the DMPS spectra within a cluster (cohesion) compared to other clusters (separation)^61^. The range of the silwidth is from 1 to −1, where 1 indicates that the elements within the cluster are have a high similarity with each other but a low similarity with the elements with the other clusters. For the DMPS data there is a tendency for the silwidth to be high for smaller cluster numbers which decreases as the cluster number is increased as the separation of clusters decreases. There will be a cluster number at which the natural clusters start to divide. This is point is judged as the cluster number where a common abrupt change in the DI and silwidth takes place, i.e. 9 to 10 clusters for the clustered daily DMPS spectra. Likewise, a similar approach was applied to cluster the hourly NSD data to determine the optimum number of clusters. However only the silwidth was used since the DI could not be calculated for the increased dataset size. Hence the largest number of clusters (13) was chosen with a value of the silwidth = 0.3 which showed the most abrupt decrease for an increase in cluster number. Both approaches ‘over-clustered’ the hourly and daily NSDs into 13 and 9 groups respectively when comparing time series of clusters. Clusters with similar time series and average NSD were thus merged yielding 6 hourly and 5 daily clusters.

### Aerosol particle number concentrations

Two different Condensation Particle Counter (CPC) were deployed to measure simultaneously particles at 3 nm and 10 nm (TSI CPC Model 3010 and TSI CPC Model 3025, respectively).

### Air mass back-trajectory cluster

Two step process of ‘over-clustering’, followed by ‘merging’, was carried out when applying k-Means to the back trajectory calculation. Using the BADC Trajectory Service, 5 day back trajectories arriving at Zepellin over the 10 year span at 00:00, 06:00, 12:00 and 18:00 were calculated arriving at 100 m altitude. The length of the back-trajectory calculation is chosen as a balance between the typical lifetime of the aerosols in the arctic troposphere, which is up to two weeks (shorter in summer and longer in winter/spring) for the accumulation-mode particles^58^, and the increasing uncertainty in the calculation the further back in time it goes. Because newly formed particles are likely to grow into cloud condensation nuclei in a day or two, we believe 5 days back-trajectories are more robust and more indicated for the present study. By making rows of 240 latitudes followed by 240 longitude values - sequenced by the half hour step calculated back from their arrival location - a 480 × 14,600 trajectory matrix was made for cluster analysis. By over clustering the trajectories into 10 groups, it was clear when plotting on a map that 5 groups could be used to describe the data.

Simple calculations were also made for each of the 5-day back trajectories using daily Arctic maps of gridded sea ice information. For each of the position alone each of the trajectories the sea ice information was logged into a file from which the exposure to sea ice of all the air masses arriving at Zeppelin over the 10 year study period could be calculated.

The Polar Stereographic map of the Northern Hemisphere classified each of 1024 × 1024 24 km grid cells as land, sea, ice or snow ice, and from this, the percentage of time each clustered back trajectory spent over each type could be calculated. The snow and ice coverage values were produced by the NOAA/NESDIS Interactive Multisensor Snow and Ice Mapping System (IMS) developed under the direction of the Interactive Processing Branch (IPB) of the Satellite Services Division (SSD)^62^. A similar calculation was repeated but using daily maps of sea ice percentage concentration measured on a 12.5 km grid. These Artic polar sterographic maps of 12.5 km resolution contained sea ice concentration from the 85 GHz channel of SSM/I on DMSP, available since 1992. The percentages assigned from these maps to each trajectory step allow a ‘spectrum’ of sea ice concentration of 5% width from 0 to 100% to be calculated for each of the trajectory clusters.

### Aerosol and gas chemical tracers

PM_10_ Sampling and Analysis PM_10_ aerosol sampling was performed at Gruvebadet station located about 50 m a.s.l. and 1 km far from the scientific village of Ny Ålesund (Svalbard Island −78.9°N, 11.9°E). The aerosol sampling performed by a TECORA Skypost sequential sampler equipped with a PM10 sampling head operating following the EN 12341 European rules. Aerosol samples were collected daily on Teflon (PALL Gelman) filters from March to September 2010, in total 151 samples were analysed^38^. MSA was determined by ion chromatography on the aqueous extract obtained from one half of each filter^38^. The Particle Soot Absorption Photometer (PSAP) is used to measure in near real time the optical extinction coefficient for absorption which can in also be used to estimate concentrations of black carbon. The resulting concentrations are reported as equivalent black carbon (EBC). EBC at daily resolution available at the NILU website data at daily resolution for the period 2001–2010. Gaseous NH_3_ and SO_2_ data were obtained at daily resolution at the NILU website data for the period 2001–2010.

### Cloud Condensation Nuclei data

Concentrations of Cloud Condensation Nuclei (CCN) have been measured continuously using a commercial available Droplet Measurement Technology (DMT) CCN counter. The CCN counter measures the number of particles large enough to act as CCN by feeding the ambient air though the chamber in which supersaturation is achieved. The CCN can scan particle number distribution as a function of supersaturation. We set the CCNC to measure concentration of CCN for 0.2, 0.4, 0.6, 0.8, 1.0 Super Saturation (SS). During the study, flow and zero-counting were checked frequently, calibrations were carried out over the years, the instrument was found stable and overall resulting data satisfactory. In this study we have select CCN values when SS is 0.4%. In total, 1120 days of sampling were obtained at hourly resolution between 01-04-2007 and 31-12-2010.

### Calculation of the Condensation Sink

The condensation sink (CS) describes how rapidly condensable vapour molecules will condense on the existing aerosol. Specifically this quantity describes the loss rate of molecules with diameter dp, diffusion coefficient D, and mean free path λv onto a distribution n(dp) (or Ni in the discrete case) of existing particles and as such, can be obtained from integrating over the particle size spectrum^63^. Calculation are described elsewhere^64^.

### Data availability

The data that support the findings of this study are available from the corresponding author on request.

## Electronic supplementary material

supporting information
