# Supplementary material for: Arctic sea ice melt leads to atmospheric new particle formation
Source: Sci Rep. 2017 Jun 12;7:3318. doi: 10.1038/s41598-017-03328-1 (PMC5468288; doi:10.1038/s41598-017-03328-1)
Supplement: Supplementary file 1 — supporting information [file 41598_2017_3328_MOESM1_ESM.pdf]

SUPPLEMENTARY INFORMATION

Arctic sea ice melt leads to atmospheric new particle formation

M. Dall´Osto, D. Beddows, P. Tunved, R. Krejci, J. Ström, H-C Hansson, Y.J. Yoon, Ki-Tae Park, S. Becagli, R. Udisti, T. Onasch, C.D. O´Dowd, R. Simo and Roy M. Harrison

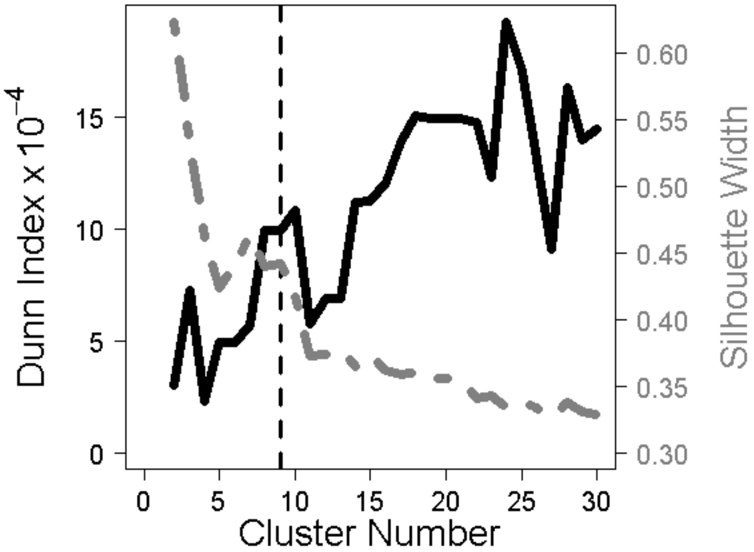

Supplementary Figure S1. Plot of Dunn Index and Silhouette Width against cluster number to identify the main cluster groups within the data. Cluster number 9 was been identified as the optimum number of clusters within the daily DMPS average number size distributions. Dashed line marks the optimum (9th) clusters.

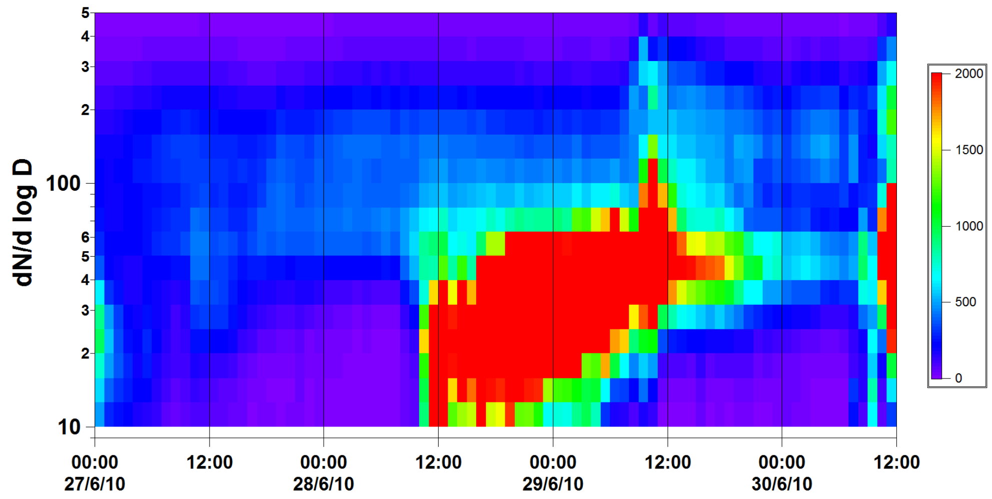

(a)

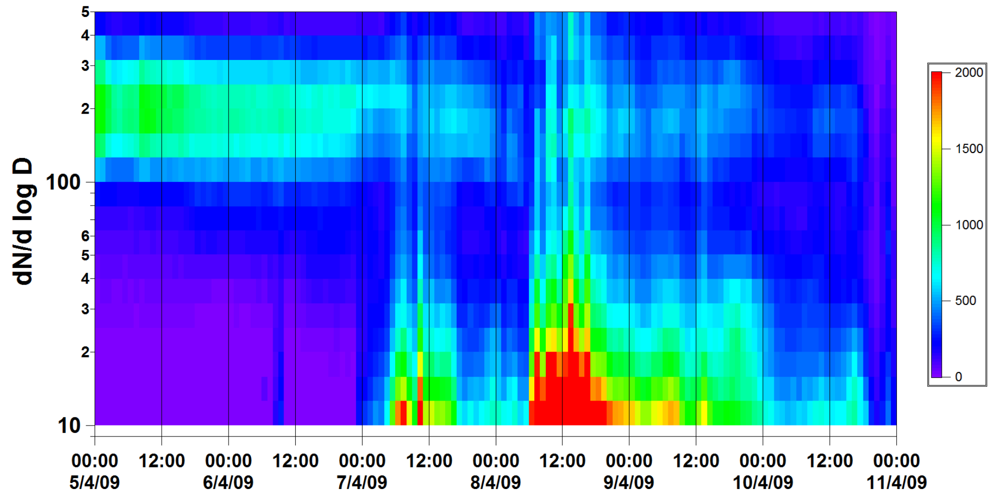

(b)

Supplementary Figure S2. Real time concentrations (cm^{-3}) data examples of (a) Nucleation event with growth and (b) nascent ultrafine particles with no subsequent growth. The total particle number concentration ($N_{10\text{-}500nm}$) was found correlated with concurrent measurements taken of total particle number concentrations with CPC3010 and CPC2025, allowing to obtain the $N_{3\text{-}10nm}$ concentrations.

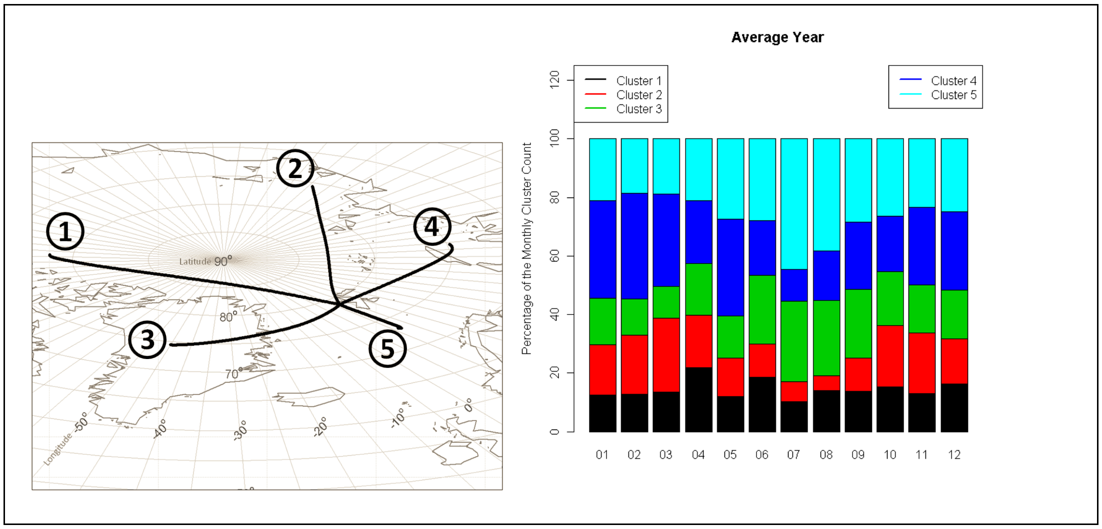

Supplementary Figure S3. Air mass back trajectory cluster analysis results (left) and annual variation of the percentage of the monthly cluster count (right). This plot was created using the R software (R Development Core Team, R i386 3.3.2; www.r-project.org).

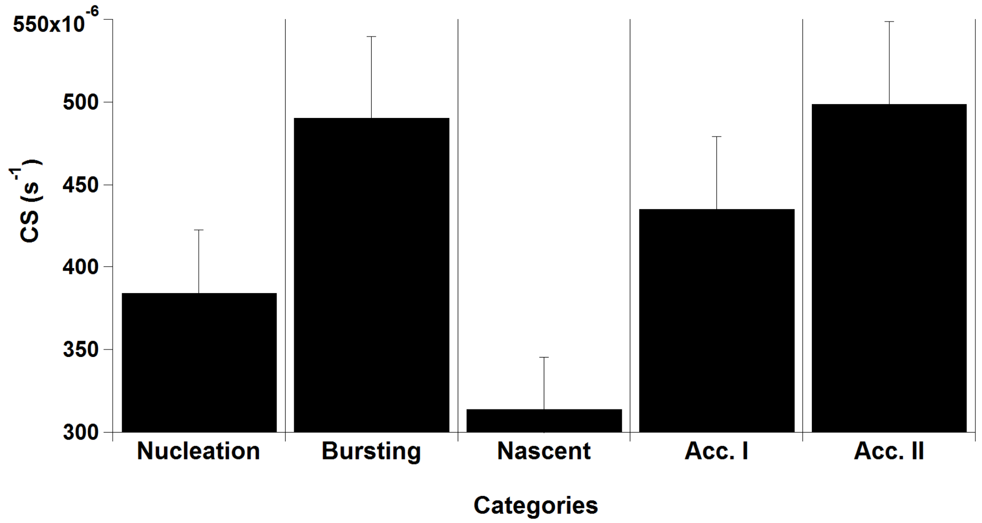

Supplementary Figure S4. Calculation of Condensational Sink (s^{-1}) for the five aerosol categories

SMPS cluster	*Air mass classification*					*Geographical region*			
	1	2	3	4	5	sea	sea ice	snow	land
	N	N	SW	E	S				
**Nucleation**	14	7	25	15	38	43	39	14	4
**Bursting**	10	6	28	19	37	43	37	17	3
**Nascent**	10	6	22	20	42	51	29	13	7
**Accumulation I**	18	24	12	31	14	14	62	23	1
**Accumlation II**	11	15	19	23	30	32	45	20	3

Supplementary Table S1. Relation of aerosol SMPS cluster analysis (Figure 1) with air mass back trajectories cluster analysis (cluster 1-5: main origin N: North, SW: South West, E: East and S: South; Figure S3) and geographical region (sea, sea ice, snow, land). Percentages for air mass classification (Figure S3) are normalized over aerosol cluster category (Figure 1). Percentages of geographical region are normalised by the period of passage over land, snow, open water and sea ice .
